# Prognostic Value of the Veno-Arterial Carbon Dioxide Difference in COVID-19-Associated Acute Hypoxemic Respiratory Failure: A Retrospective Cohort Study

**DOI:** 10.3390/diagnostics16213522

**Published:** 2026-10-27

**Authors:** Keitiane Kaefer, Sara Fratino, Rocio Rimachi, Antonella Cudia, Rachid Attou, Pierre Foulon, Charalampos Pierrakos, Daniel De Backer

**Affiliations:** 1 Intensive Care Department, Brugmann University Hospital, Université Libre de Bruxelles, 1020 Brussels, Belgium; 2 Intensive Care Department, CHIREC Hospitals, Université Libre de Bruxelles, 1160 Brussels, Belgiumrocio.rimachi@gmail.com (R.R.);

**Keywords:** P_va_CO_2_ gap, tissue perfusion, carbon dioxide kinetics, Haldane effect, hypoxemia, prognosis, survival, central venous oxygen saturation, ARDS

## Abstract

**Background/Objectives:** In acute hypoxemic respiratory failure, hyperventilation and alterations in oxygenation can influence CO_2_ kinetics via the Haldane effect, potentially modifying the venous-to-arterial carbon dioxide partial pressure gradient (PvaCO_2_) irrespective of tissue perfusion, thereby complicating its prognostic significance. This study assessed the predictive importance of PvaCO_2_ in critically ill patients experiencing acute respiratory failure linked to COVID-19. **Methods:** This retrospective bicentric study included critically ill patients with hypoxemic COVID-19 (P_a_O_2_/FiO_2_ < 300 mmHg) admitted to the intensive care unit (ICU) during the first and second waves of the Belgium epidemic, with at least one PvaCO_2_ assessment within 5 days of admission. PvaCO_2_ was assessed early after ICU admission using paired arterial and central venous blood gases. The highest daily value was registered. The first registered value was considered as a baseline. The primary outcome was ICU death within 28 days. **Results:** Among 300 ICU admissions, 136 patients met inclusion criteria, with PvaCO_2_ measured within 48 h in 76%. Median P_a_O_2_/FiO_2_ was 123 (79–176) mmHg, with no significant differences in baseline severity between patients with normal or abnormal PvaCO_2_. Lactate levels were generally normal, and inotrope use was uncommon. Early abnormal PvaCO_2_ was not significantly associated with ICU death within 28 days in the competing-risk analysis (sHR 1.69, 95% CI 0.86–3.33; *p* = 0.13), while complementary Bayesian analysis indicated an 89.7% posterior probability of increased hazard. When stratified by hypoxemia severity—severe (PaO_2_/FiO_2_ ≤ 100 mmHg), moderate (>100–200 mmHg), and mild (>200 mmHg)—a stronger exploratory signal related to ICU mortality was observed in moderate hypoxemia; however, the formal interaction did not reach statistical significance (Pinteraction = 0.075). **Conclusions:** Early PvaCO_2_ was not significantly associated with ICU death within 28 days in patients with predominantly hypoxemic respiratory failure without major baseline circulatory shock. Exploratory subgroup analyses suggested that its association with outcome may vary according to arterial and central venous oxygenation. This hypothesis warrants targeted evaluation in future prospective studies.

## 1. Introduction

The venous-to-arterial carbon dioxide partial pressure difference (PvaCO_2_), calculated as PvCO_2_—PaCO_2_ obtained from simultaneous central venous and arterial blood gas analyses, is widely used as a bedside marker of cardiac output adequacy relative to metabolic demand [1,2,3]. According to the Fick principle, PvaCO_2_ is inversely related to cardiac output and influenced by CO_2_ production, assuming a near-linear relationship between CO_2_ content and partial pressure [4]. Elevated PvaCO_2_ values, commonly defined as >6 mmHg, have been associated with impaired global perfusion, microcirculatory dysfunction, and increased mortality in critically ill patients [5]. Accordingly, PvaCO_2_ has emerged as a practical and clinically meaningful marker of global tissue perfusion.

However, the physiological interpretation of PvaCO_2_ may be substantially more complex in the setting of acute hypoxemic respiratory failure. Hyperventilation can rapidly lower arterial PCO_2_ while central venous CO_2_ decreases more slowly, resulting in an apparent widening of the PvaCO_2_ gradient even without true circulatory impairment [6]. In parallel, hypoxemia alters CO_2_ transport through the Haldane effect, whereby deoxygenated hemoglobin binds CO_2_ more avidly, potentially reducing arterial CO_2_ for a given CO_2_ content [7]. Conversely, improvements in oxygenation or increases in cardiac output may raise central venous oxygen saturation, attenuate the Haldane effect, and increase venous CO_2_. Together, these mechanisms suggest that PvaCO_2_ may be influenced by respiratory and oxygenation-related factors independent of tissue perfusion.

Despite these theoretical concerns, the clinical value of PvaCO_2_ in acute respiratory failure remains uncertain, as isolated respiratory failure is uncommon, making its independent impact difficult to evaluate. COVID-19–related acute respiratory failure represents a relevant clinical model, as profound hypoxemia frequently coexists with preserved or even elevated cardiac output [8]. In addition, particularly during the early phase of the disease, the severity of hypoxemia may be disproportionate to the impairment in respiratory-system mechanics [9]. COVID-19 may also be associated with increased dead-space ventilation and pulmonary perfusion abnormalities, which may influence PaCO_2_ independently of changes in systemic perfusion and not necessarily in proportion to the severity of hypoxemia [10]. In this setting, it remains unclear whether an elevated PvaCO_2_ reflects persistent tissue hypoperfusion superimposed on respiratory failure or is merely driven by acute oxygenation-related changes in CO_2_ kinetics. Accordingly, this study aimed to evaluate the association between early PvaCO_2_ and mortality in critically ill COVID-19 patients and to assess its potential value for bedside prognostic stratification across different degrees of hypoxemia. We hypothesized that elevated early PvaCO_2_ would be associated with a higher risk of ICU death within 28 days, and this association may differ according to the severity of hypoxemia.

## 2. Methods

### 2.1. Design

We retrospectively reviewed patients with COVID–19 confirmed by reverse transcriptase polymerase chain reaction (RT–PCR) who were treated in two independent large ICUs in Brussels, Belgium (Brugmann University Hospital and CHIREC Hospitals), between 1 March 2020 and 31 December 2020. PvaCO_2_ evaluation was performed as routine practice in both centers in patients equipped with a central line and arterial line. The database was accessed for research purposes between 12 November 2024 and 31 May 2025.

### 2.2. Patients

Patients were included if a PvaCO_2_ measurement was obtained within the first 5 days after admission to the ICU. Patients were excluded if they were evaluated while supported with extracorporeal membrane oxygenation (ECMO) during the first 5 days, if their outcome was unknown, and/or if they had P_a_O_2_/FiO_2_ ≥ 300 mmHg at admission.

### 2.3. Data Collected

Data extracted from patient charts included: demographic characteristics; the Simplified Acute Physiology Score (SAPS) II and Sequential Organ Failure Assessment (SOFA) score calculated using the worst values recorded during the first 24 h after ICU admission; ventilator, blood gas, and laboratory analyses during the first 5 days of admission; complications; and procedures during hospitalization. During the COVID-19 period, patients with hypoxemic respiratory failure were not always equipped with a central venous catheter directly at ICU admission. In both participating ICUs, central venous catheter placement followed standard clinical indications, which included, among others, hemodynamic instability requiring vasoactive or inotropic support or repeated fluid resuscitation. Vasoactive and inotropic agents, including norepinephrine, were administered exclusively through central venous access rather than peripheral venous lines. As soon as central venous access was obtained, a venous blood gas was sampled. PvaCO_2_ was routinely assessed as part of standard care when both arterial and central venous access became available, irrespective of hemodynamic status. The worst value of PvaCO_2_ for each day was used for the analysis. A value higher than 6 mmHg was considered abnormal. The first available PvaCO_2_ assessment within 5 days of ICU admission was considered the baseline PvaCO_2_ assessment. Paired arterial and central venous blood-gas variables were measured concomitantly at this assessment; subsequent daily measurements were used only for the longitudinal analyses. Ventilatory and other physiological variables reported in relation to the first PvaCO_2_ assessment were obtained from the closest available measurement within 1 h of that assessment. Norepinephrine and dobutamine use referred to the day of the first PvaCO_2_ assessment. The maximum norepinephrine dose was defined as the highest dose recorded during the first 5 ICU days among patients who received norepinephrine; patients with no norepinephrine exposure (dose = 0 mcg/kg/min) were not included in the dose analysis. CRP and D-dimer values were obtained from routine laboratory testing performed on the same calendar day as the first PvaCO_2_ assessment. Hemoglobin concentration, arterial and central venous oxygen partial pressures (P_a_O_2_ and P_v_O_2_), and oxygen saturations (S_a_O_2_ and S_cv_O_2_) were measured concomitantly from paired arterial and central venous blood gas analyses obtained at the same time point. Using standard formulas, arterial (C_a_O_2_) and venous (C_v_O_2_) oxygen content were calculated, and the PvaCO_2_/C_av_O_2_ ratio was derived. The P_a_O_2_/FiO_2_ ratio was calculated at the time of evaluation, with FiO_2_ corresponding to the fraction of inspired oxygen delivered to the patient. At the time of PaO_2_/FiO_2_ assessment, all patients were receiving either high-flow nasal oxygen or invasive mechanical ventilation; FiO_2_ was obtained directly from the device setting.

### 2.4. Outcomes

The primary outcome was ICU death within 28 days. Secondary outcomes were ICU death within 90 days and the longitudinal evolution of PvaCO_2_, ScvO_2_, PvaCO_2_/CavO_2_, and PaO_2_/FiO_2_ during the four days following the first evaluation.

### 2.5. Analysis Plan

No formal power calculation was performed for this exploratory analysis. Rather, all patients that fulfilled the inclusion and exclusion criteria were included. Demographic, clinical, and outcome variables were presented as percentages for categorical variables and medians with interquartile ranges (IQRs) for continuous variables. Fisher’s exact and Wilcoxon’s rank sum tests were applied as appropriate. Missing data were not imputed, and analyses were performed using available observations for each variable.

For ICU mortality, alive ICU discharge was treated as a competing event. Cumulative incidence functions were estimated and compared using Gray’s test, and Fine–Gray regression was used to estimate subdistribution hazard ratios (sHRs) with 95% confidence intervals. ICU admission was used as time zero for the primary analyses of 28- and 90-day ICU mortality. This analysis used ICU admission as the anchor. The interval between ICU admission and the first PvaCO_2_ measurement contributed to follow-up, and patients were classified according to the first PvaCO_2_ value subsequently obtained. To address variability in the timing of exposure ascertainment, two complementary sensitivity analyses were performed. First, the first PvaCO_2_ assessment was used as time zero, with follow-up extending for 28 days after that measurement. Second, the primary admission-anchored analysis was repeated after restriction to patients whose first PvaCO_2_ assessment was obtained within 48 h of ICU admission. Adjusted Fine–Gray models, including SAPS II and study center, were fitted as sensitivity analyses. Given the limited number of ICU deaths, a parsimonious adjustment strategy was used to reduce the risk of overfitting.

Additionally, a Bayesian Cox proportional-hazards analysis was performed for 28-day ICU mortality. A diffuse Normal(0, 5) prior was assigned to the PvaCO_2_ coefficient on the log-hazard-ratio scale. Posterior inference was obtained using four Markov Chain Monte Carlo chains with 4000 iterations per chain, including 1000 warm-up iterations, yielding 12,000 post-warm-up draws. Sampling was performed using the No-U-Turn Sampler with an adapt_delta of 0.95. Convergence and sampling performance were assessed using R-hat, bulk and tail effective sample sizes, trace plots, divergent transitions, and maximum-treedepth diagnostics. The proportional-hazards assumption was assessed using Schoenfeld residuals. Sensitivity analyses were performed using Normal(0, 1) and Normal(0, 0.5) priors on the log-hazard- ratio scale. Results are reported as posterior median hazard ratios, 95% credible intervals, and posterior probabilities of HR > 1. As an exploratory complementary analysis, a pairwise win-ratio approach was used to compare patients with low versus high PvaCO_2_. All between-group patient pairs were compared according to a three-level clinically ordered 28-day outcome hierarchy: ICU death within 28 days, persistent ICU stay at day 28, and alive ICU discharge before day 28. This hierarchy was chosen to reflect increasing clinical severity, with death considered the most clinically consequential outcome, persistent ICU stay representing ongoing critical illness and dependence on intensive care, and alive ICU discharge representing the most favorable clinical course. Pairs were adjudicated according to the first differing outcome category, whereas pairs with the same category were classified as ties. Time to death and time to ICU discharge within categories were not used for further adjudication. The win ratio was calculated as the number of wins in the low-PvaCO_2_ group divided by the number of wins in the high-PvaCO_2_ group. A 95% confidence interval was derived from 1000 patient-level bootstrap resamples performed separately within each group. Sensitivity analyses were performed for the following predefined subgroups: (a) severe hypoxemia (P_a_O_2_/FiO_2_ ≤ 100 mmHg), (b) moderate hypoxemia (P_a_O_2_/FiO_2_ 101 to 200 mmHg) and (c) mild hypoxemia (P_a_O_2_/FiO_2_ > 200 mmHg). A post hoc analysis stratified patients according to baseline ScvO_2_ (<70% vs. ≥70%) to explore whether venous oxygenation modified the association between PvaCO_2_ and ICU mortality, given the physiological interplay between arterial oxygenation, ScvO_2_, and CO_2_ transport.

Alluvial diagrams were created to illustrate the trajectory of PvaCO_2_ over four days, stratified by PvaCO_2_ categories (0–6 mmHg or >6 mmHg) and patient outcomes (alive or deceased). Longitudinal changes in PvaCO_2_, S_cv_O_2_, the PvaCO_2_/C_av_O_2_ ratio, and the P_a_O_2_/FiO_2_ ratio were analyzed using linear mixed-effects models. The first measurement was used as the reference for all subsequent measurements. A random intercept was included for each subject to account for between-subject variability in baseline values. Fixed effects were estimated and reported with 95% confidence intervals (CIs). Given the retrospective nature of the study and the possibility of informative missingness, these longitudinal analyses were considered exploratory.

All analyses were performed in R through the R-studio interface (www.r-project.org, R version 4.2.1). *p*-values < 0.05 were considered significant.

## 3. Results

From 300 ICU admissions during the study period, 136 patients met the study criteria (Supplementary Figure S1). PvaCO_2_ was measured within 48 h in 103 patients (75.7%). The median P_a_O_2_/FiO_2_ of the patients was 123 (79–176)mmHg. Patient characteristics (Table 1) revealed no significant differences in disease severity across groups of patients with normal or abnormal PvaCO_2_. The lactate levels were within normal range in both groups and very few patients were supported by inotropes.

### 3.1. Association of PvaCO_2_ with ICU Mortality

**Figure 1:**
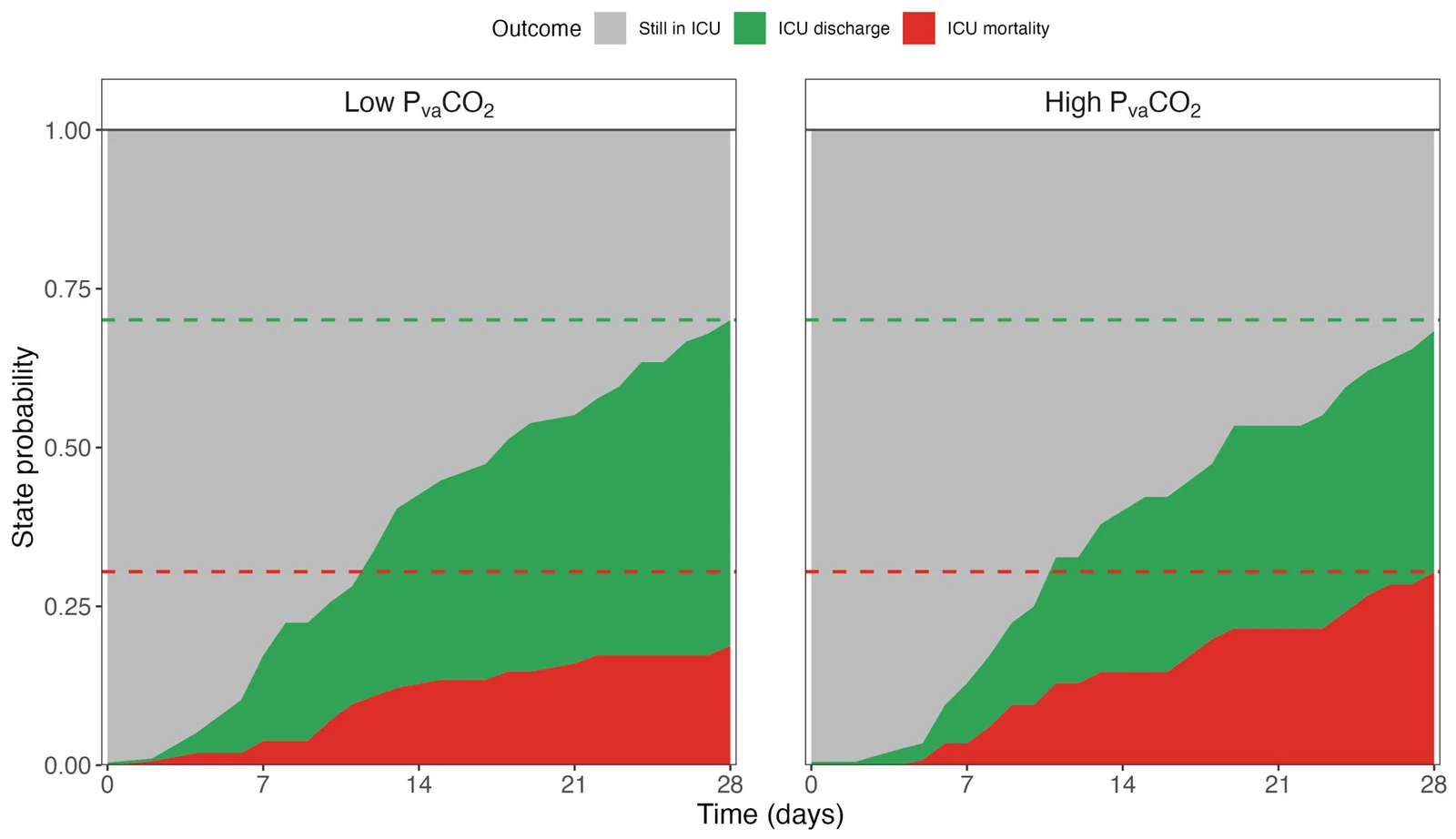
Cumulative incidence of ICU death and alive ICU discharge up to 28 days after ICU admission according to first available PvaCO_2_ Patients were classified as having low PvaCO_2_ (≤6 mmHg; n = 78, 15 ICU deaths within 28 days) or high PvaCO_2_ (>6 mmHg; n = 58, 18 ICU deaths within 28 days). Alive ICU discharge was treated as a competing event. The stacked areas represent the probabilities of ICU death, alive ICU discharge, and remaining in the ICU over time. Horizontal dashed lines indicate the maximum observed cumulative incidence of ICU death (red) and alive ICU discharge (green) across the PvaCO_2_ groups.

In the admission-anchored primary analysis, in which patients were classified according to their first available PvaCO_2_ measurement, elevated PvaCO_2_ was not significantly associated with ICU death within 28 days in the primary competing-risk analysis (sHR 1.69, 95% CI 0.86–3.33; *p* = 0.13, Figure 1). A similar nonsignificant pattern was observed for ICU death within 90 days (Supplementary Figure S2). Crude mortality was higher among patients with abnormal PvaCO_2_ than among those with normal values (31% vs. 19%, *p* = 0.15). The first PvaCO_2_ assessment was obtained within 48 h of ICU admission in 103/136 patients (75.7%). No patient died within 48 h of ICU admission before central venous catheter placement and the first PvaCO_2_ measurement. Results were directionally similar when follow-up was anchored at the first PvaCO_2_ assessment and in the subgroup assessed within 48 h, although estimates remained imprecise after adjustment (Supplementary Table S1). In the primary 28-day analysis from ICU admission, the unadjusted association was attenuated after adjustment for SAPS II and study centre, with wide confidence intervals (Supplementary Table S1).

In complementary Bayesian survival analysis, abnormal PvaCO_2_ was associated with a posterior hazard ratio of 1.55 (95% credible interval 0.78–3.08) for 28-day ICU mortality, corresponding to an 89.7% posterior probability of an increased hazard. Estimates remained directionally consistent in sensitivity analyses using more informative priors, with modest attenuation toward the null (Supplementary Table S2). Consistently, the exploratory win-ratio analysis, 4524 between-group pairs were compared, yielding 1854 wins for the low-PvaCO_2_ group, 1106 wins for the high-PvaCO_2_ group, and 1564 ties. The resulting win ratio was 1.68 (bootstrap 95% CI 1.00–3.03).

### 3.2. Association of PvaCO_2_ with Hypoxemia Severity and ICU Mortality

**Figure 2:**
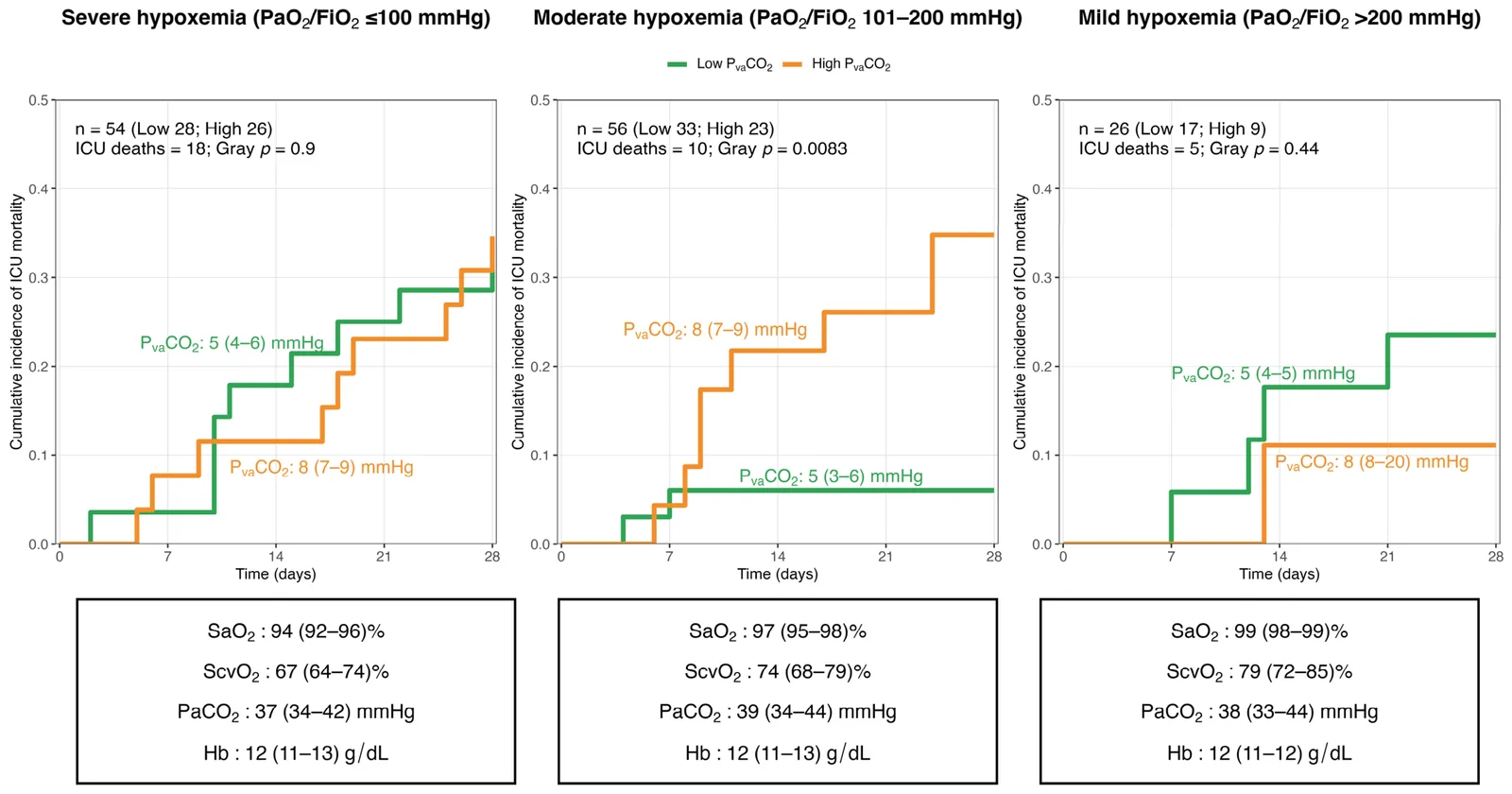
Cumulative incidence of ICU death within 28 days according to first available PvaCO_2_ and severity of hypoxemia. Patients were stratified into severe (PaO_2_/FiO_2_ ≤ 100 mmHg), moderate (PaO_2_/FiO_2_ 101–200 mmHg), and mild hypoxemia (PaO_2_/FiO_2_ > 200 mmHg). Green lines represent patients with low PvaCO_2_ (≤6 mmHg) and orange lines patients with high PvaCO_2_ (>6 mmHg). The number of patients, ICU deaths, and corresponding *p* values are reported within each panel. Baseline SaO_2_, ScvO_2_, PaCO_2_, and hemoglobin values for each hypoxemia stratum are shown below the corresponding panel. PaO_2_/FiO_2_, ratio of arterial oxygen partial pressure to fractional inspired oxygen; PvaCO_2_, central venous-to-arterial carbon dioxide tension difference; SaO_2_, arterial oxygen saturation; ScvO_2_, central venous oxygen saturation; PaCO_2_, arterial carbon dioxide partial pressure; Hb, hemoglobin.

When stratified by hypoxemia severity, the association between elevated PvaCO_2_ and ICU mortality appeared strongest in the moderate-hypoxemia subgroup (sHR 6.68, 95% CI 1.36–32.69), whereas no clear association was observed in severe (sHR 1.06, 95% CI 0.44–2.57) or mild hypoxemia (sHR 0.44, 95% CI 0.05–3.86) (Figure 2) However, formal interaction testing did not reach statistical significance (Pinteraction = 0.075); therefore, this pattern was considered exploratory (Supplementary Table S3). Across hypoxemia categories, the proportion of patients with abnormal PvaCO_2_ (>6 mmHg) decreased with improving oxygenation, while the relative frequency of markedly elevated PvaCO_2_ values (>10 mmHg) increased in patients with less severe hypoxemia (Supplementary Figure S3).

### 3.3. Association of PvaCO_2_ with Mortality in Relation to S_cv_O_2_ Levels

**Figure 3:**
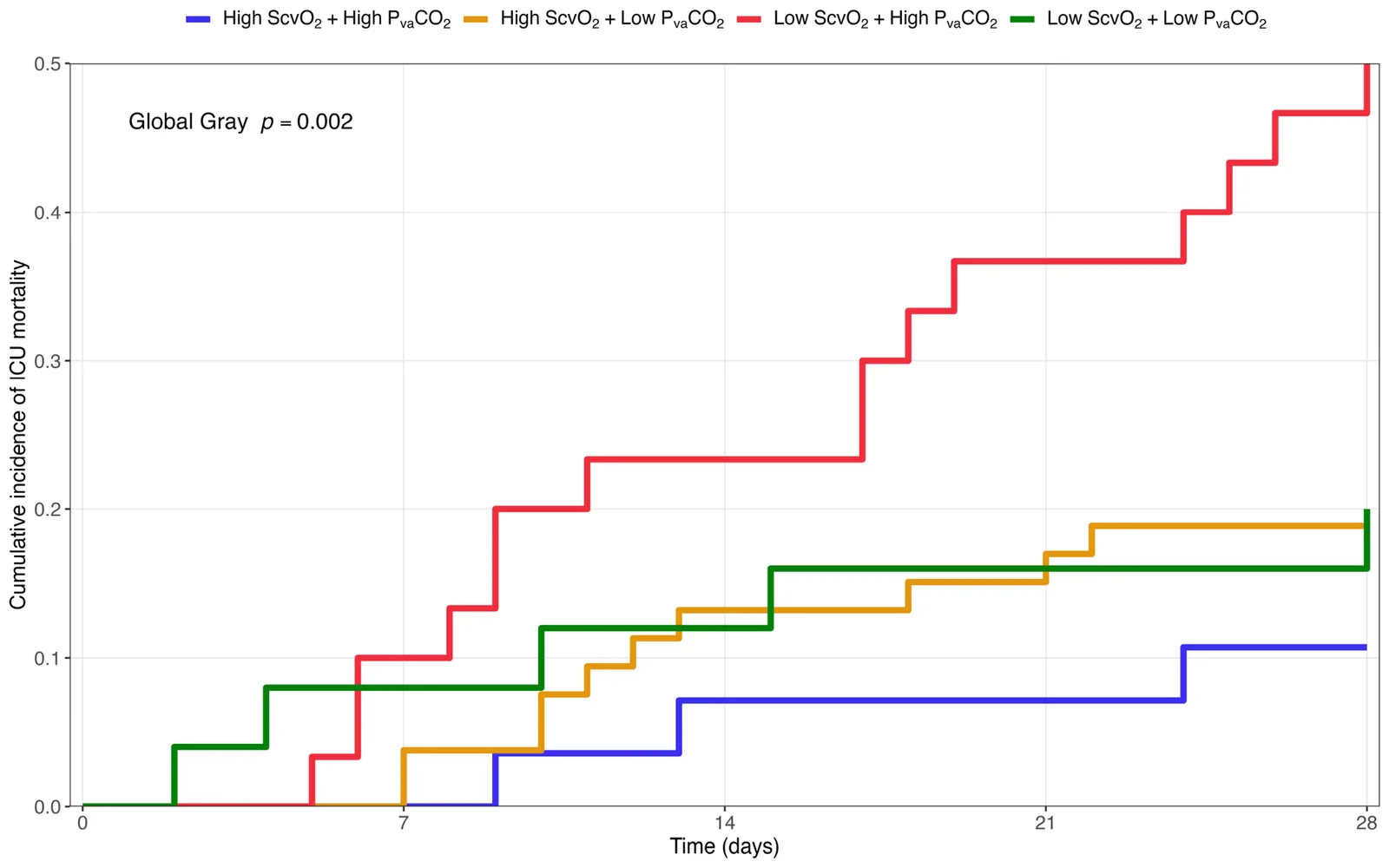
Cumulative incidence of ICU death within 28 days according to baseline ScvO_2_ and PvaCO_2_ categories. Patients were classified into four groups: ScvO_2_ ≥ 70% with high PvaCO_2_ (>6 mmHg; n = 28, 3 ICU deaths), ScvO_2_ ≥ 70% with low PvaCO_2_ (≤6 mmHg; n = 53, 10 ICU deaths), ScvO_2_ < 70% with high PvaCO_2_ (>6 mmHg; n = 30, 15 ICU deaths), and ScvO_2_ < 70% with low PvaCO_2_ (≤6 mmHg; n = 25, 5 ICU deaths). The global Gray test comparing cumulative incidence functions across the four groups was *p* = 0.0021. Abbreviations: ScvO_2_, central venous oxygen saturation; PvaCO_2_, central venous-to-arterial carbon dioxide tension difference; ICU, intensive care unit.

In the post hoc analysis, elevated PvaCO_2_ was associated with higher ICU mortality among patients with ScvO_2_ < 70% (sHR 2.89, 95% CI 1.04–7.98), whereas no clear association was observed among patients with ScvO_2_ ≥ 70% (sHR 0.54, 95% CI 0.15–1.93). Elevated PvaCO_2_ was associated with lower ICU survival in patients with reduced S_cv_O_2_, whereas survival curves largely overlapped in patients with S_cv_O_2_ ≥ 70% (Figure 3 and Figure S4). The interaction between PvaCO_2_ category and ScvO_2_ category was statistically significant (Pinteraction = 0.044; Supplementary Table S4). Given the post hoc nature of this analysis, these findings should be considered exploratory.

### 3.4. PvaCO_2_ Trajectory in Surviving and Deceased Patients

**Figure 4:**
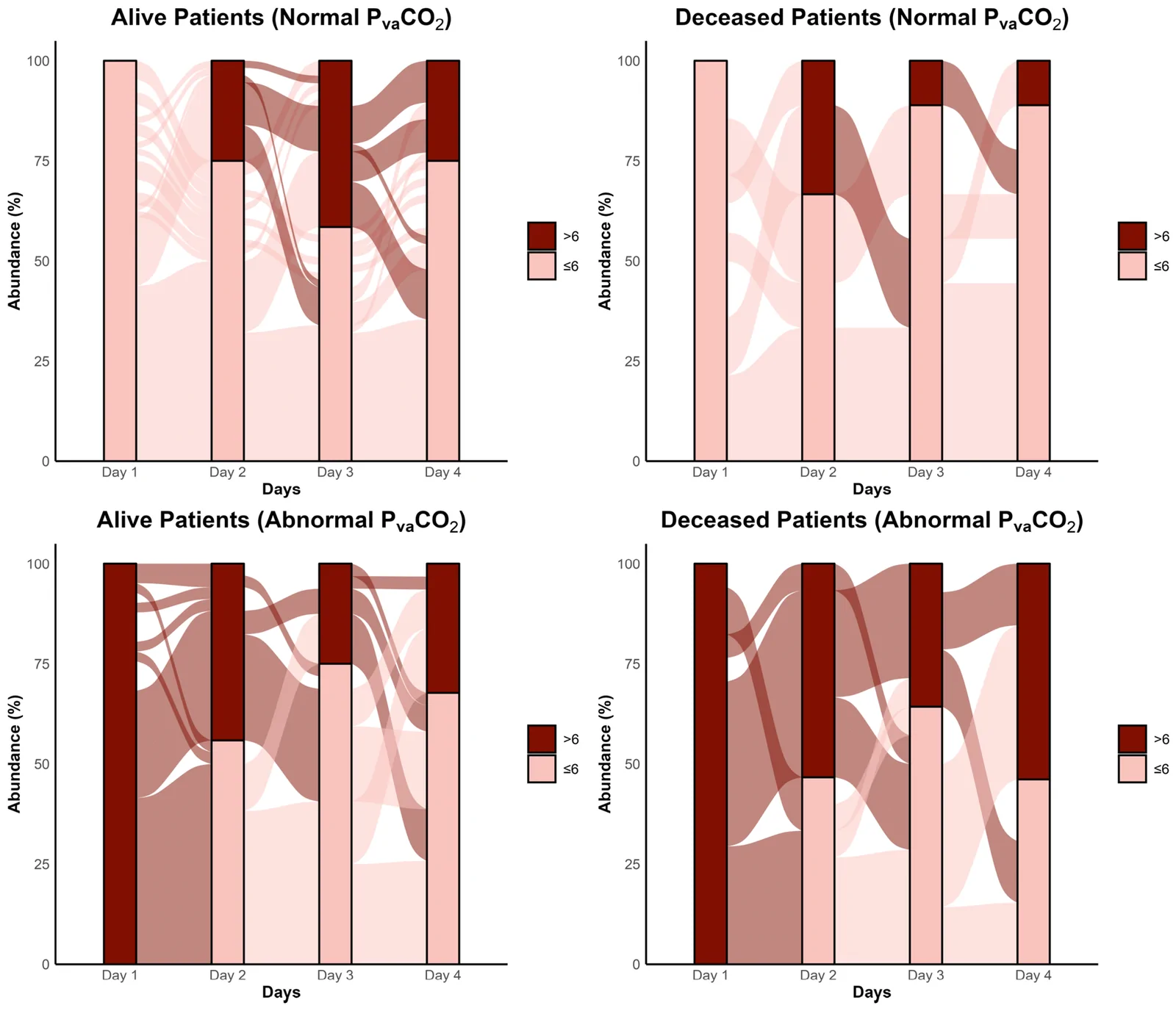
Dynamic changes in venous-to-arterial carbon dioxide tension (PvaCO_2_) over the first four days after the initial evaluation in patients who survived or died up to 28 days after intensive care admission. PvaCO_2_ is categorized as ≤6 mmHg (light pink) or >6 mmHg (dark red). Transitions between categories are represented by the flows between bars over the four days.

PvaCO_2_ trajectories differed according to baseline values and outcome. Patients with low baseline PvaCO_2_ rarely transitioned to abnormal values during follow-up, irrespective of survival, whereas elevated baseline PvaCO_2_ persisted more frequently in patients who died (Figure 4). Notably, longitudinal physiological evolution differed by baseline PvaCO_2_ status. Among patients with elevated baseline PvaCO_2_, non-survivors showed improvement in oxygenation without relevant changes in other parameters, while among patients with low baseline PvaCO_2_, non-survival was characterized by a progressive increase in the PvaCO_2_/C_av_O_2_ ratio despite stable PvaCO_2_ values and not any improvement in oxygenation (Supplementary Figures S5 and S6). ICU discharge and death accounted for only a small proportion of unavailable measurements during the four-day observation period (Supplementary Table S5).

## 4. Discussion

The results of this study can be summarized as follows: (1) in critically ill patients with acute respiratory failure, early elevation of PvaCO_2_ was inconsistently associated with ICU mortality, depending on analytical approaches in the overall population; (2) the relationship between PvaCO_2_ and outcome varied according to hypoxemia severity and venous oxygenation status, with prognostic separation observed in patients with moderate hypoxemia and reduced S_cv_O_2_; and (3) longitudinal analyses identified distinct PvaCO_2_ trajectories according to baseline values and outcome, with persistent elevation more frequently observed in non-survivors, including patients who exhibited concurrent improvement in oxygenation over time.

To our knowledge, this is the first study to comprehensively evaluate the early prognostic value of PvaCO_2_ in critically ill patients with acute hypoxemic respiratory failure. By focusing on patients admitted during the COVID-19 pandemic, we examined a clinically homogeneous population with predominantly hypoxemic respiratory failure. Baseline lactate levels were generally normal, and vasoactive or inotropic support was limited. Importantly, most patients in the present cohort exhibited preserved acid–base status, limiting the confounding influence of metabolic acidosis on PvaCO_2_ interpretation [11]. Inclusion across a broad range of hypoxemia severities enabled meaningful subgroup analyses, clarifying how oxygenation status influences the prognostic relevance of PvaCO_2_. Importantly, the study design allowed longitudinal assessment of PvaCO_2_ alongside other readily available bedside metabolic parameters during the first four ICU days using routine measurements. The bicentric design reduces reliance on a single-centre experience and provides some reassurance regarding the consistency of the findings across two independent ICUs, although generalizability remains limited by the selected COVID-19 population and treatment era. Overall, this study provides a pragmatic framework for early and longitudinal evaluation of PvaCO_2_ in hypoxemic ICU patients.

Our study evaluated the prognostic value of an early PvaCO_2_ measurement in relation to mortality, consistent with prior studies in diverse ICU populations that have yielded heterogeneous results, with some identifying a prognostic signal [12,13,14,15,16,17,18,19] while others found no significant association with mortality [12,20,21,22,23]. In our cohort, the primary competing-risk analysis did not demonstrate a statistically significant association between elevated PvaCO_2_ and ICU death within 28 days. Complementary Bayesian and win-ratio analyses showed directionally consistent signals toward poorer outcomes with elevated PvaCO_2_; however, these findings remained uncertain, with interval estimates including the null value, and should therefore be considered exploratory rather than confirmatory. Differences across analyses may reflect residual confounding, limited statistical power, and differences in model assumptions and estimands. Importantly, our population was characterized by severe hypoxemic respiratory failure without major macrohemodynamic disturbances. Hypoxemia and oxygenation-related interventions may have influenced both arterial and venous PCO_2_, thereby affecting outcome associations. Therefore, these findings emphasize the need to interpret PvaCO_2_ in the context of oxygenation severity in critically ill patients with respiratory failure.

In contrast to previous studies that repeatedly assessed PvaCO_2_ over short intra-day intervals [3,15,17,20,24,25,26,27], we evaluated its day-to-day evolution using daily measurements. Frequent repeated measurements are labor-intensive and prone to physiological noise related to ventilation, acid–base balance, and oxygenation [28]. In our exploratory analysis, distinct patterns of PvaCO_2_ evolution emerged according to baseline values, with persistent elevation showing prognostic relevance only in patients with initially increased PvaCO_2_. Patients with high versus low baseline PvaCO_2_ exhibited distinct trajectories of other metabolic parameters, suggesting that adverse outcomes in patients with low PvaCO_2_ may be driven by pathophysiological processes different from those predominating in patients with elevated PvaCO_2_. Overall, these findings refine the clinical interpretation of early PvaCO_2_ and support its use in identifying distinct physiological phenotypes.

In clinical practice, PvaCO_2_ and S_cv_O_2_ are concomitantly available parameters that together inform tissue perfusion. While PvaCO_2_, derived from the Fick principle, primarily reflects cardiac output relative to CO_2_ production, S_cv_O_2_ is additionally influenced by oxygenation status, tissue oxygen extraction, and shunting [4,29]. Accordingly, when S_cv_O_2_ is normal or elevated, PvaCO_2_ may help identify residual hypoperfusion [30]; however, previous studies have reported inconsistent additional prognostic value in this setting [17,20,22,24,31]. We hypothesize that the Haldane effect partly explains this variability, particularly in acute respiratory failure. In our cohort, most patients exhibited S_cv_O_2_ values around 70%, with supranormal values frequently observed in mild hypoxemia. Notably, the prognostic value of PvaCO_2_ was preserved only in moderate hypoxemia and attenuated in mild hypoxemia with supranormal S_cv_O_2_ and in severe hypoxemia. In these contexts, arterial hypoxemia or venous relative hyperoxemia may alter CO_2_–hemoglobin binding, increasing PvaCO_2_ independently of tissue hypoperfusion. These findings emphasize the need to interpret PvaCO_2_ alongside both arterial oxygenation and S_cv_O_2_ and warrant validation in broader ICU populations.

This study has several limitations that should be acknowledged. First, no formal sample size calculation was performed, as we elected to include all eligible patients with COVID-19 admitted during the study period. Although conventional frequentist analyses did not demonstrate statistically significant associations, the possibility of a type II error cannot be excluded, particularly given the sample size and the consistent directional signal toward worse outcomes in patients with elevated PvaCO_2_. Secondary Bayesian, win-ratio, and subgroup analyses provided directionally supportive signals, but were exploratory and not adjusted for multiplicity. The Bayesian credible interval included the null value, further underscoring the uncertainty of this finding. These analyses therefore do not overcome the possibility of type II error or establish a definitive association, but they may help inform the design of future adequately powered studies. Importantly, the absence or even inversion of prognostic separation in patients with severe and mild hypoxemia argues against a simple power-related explanation and supports a context-dependent physiological effect. Second, the limited number of ICU deaths constrained our ability to explore more flexible nonlinear effect-modification models, such as restricted cubic spline interactions between PvaCO_2_ and PaO_2_/FiO_2_. These exploratory findings may nevertheless help inform the design and hypotheses of future adequately powered studies. Third, because the first PvaCO_2_ assessment could occur up to 5 days after ICU admission, residual temporal heterogeneity in exposure ascertainment cannot be excluded despite sensitivity analyses using the first assessment as time zero and restricting measurements to within 48 h. Accordingly, the admission-anchored primary analysis should not be interpreted as evaluating a PvaCO_2_ value available at ICU admission in every patient, since clinical evolution and treatment before the first measurement may have influenced both the timing of PvaCO_2_ assessment and its measured value. Fourth, the retrospective design and reliance on routinely collected clinical data may introduce selection bias. Nearly half of otherwise eligible patients lacked PvaCO_2_ measurements, primarily due to the absence of central venous catheter placement, which may have influenced patient selection. Fifth, we did not collect data on serum bicarbonate concentration or body temperature, both of which may affect CO_2_ kinetics and PvaCO_2_ interpretation. Sixth, cardiac output was not directly measured, limiting our ability to relate PvaCO_2_ changes to actual hemodynamic alterations, and dead space fraction was not quantified. Finally, as an observational study, causal inference is not possible. Generalizability is limited to critically ill patients with COVID-19-associated hypoxemic respiratory failure in whom paired arterial and central venous blood gases were available, and extrapolation to non-COVID respiratory failure requires further evaluation. Nevertheless, this specific clinical setting provides a relevant physiological model for examining PvaCO_2_ when oxygenation, ventilation, pulmonary perfusion, and CO_2_ kinetics are simultaneously altered, and the present findings may therefore inform hypothesis generation and future prospective studies.

## 5. Conclusions

In this cohort of critically ill patients with COVID-19-associated acute hypoxemic respiratory failure, the first available PvaCO_2_ measurement obtained within the first 5 days of ICU admission was not significantly associated with ICU death within 28 days in the admission-anchored primary analysis. Exploratory analyses suggested that the association between PvaCO_2_ and outcome may vary according to arterial and central venous oxygenation. Although these findings cannot be directly generalized to non-COVID respiratory failure, COVID-19 provides a distinctive clinical and physiological model in which the interaction between oxygenation, ventilation, and CO_2_ kinetics can be examined under real-world conditions. These observations should therefore be considered hypothesis-generating and may help inform prospective studies evaluating PvaCO_2_ in broader forms of acute hypoxemic respiratory failure. Future prospective studies across broader ICU populations should further investigate the interactions among PvaCO_2_, arterial oxygenation, and ScvO_2_ and clarify the conditions under which PvaCO_2_ retains physiological and prognostic relevance.

## Figures and Tables

**Table 1:** Patient characteristics according to PvaCO_2_ category. PvaCO_2_ category was defined according to the first available PvaCO_2_ assessment within 5 days of intensive care unit (ICU) admission.

	PvaCO_2_ ≤ 6 mmHg	Available n/N	PvaCO_2_ > 6 mmHg	Available n/N	*p* Values
Number of patients	78		58		
Day of the first evaluation	2 (1–3)	78/78	2 (1–2)	58/58	0.853
Demographics					
Age (years), IQR	60 (51–69)	78/78	67 (59–74)	58/58	0.003
Female (%)	35 (45)	78/78	19 (33)	58/58	0.153
Weight (kg), IQR	83 (75–95)	76/78	84 (71–94)	56/58	0.700
Height (cm), IQR	170 (164–176)	75/78	173 (165–178)	55/58	0.273
BMI (kg/m^2^), IQR	28.4 (26.3–32.1)	75/78	27.8 (24.8–32.1)	55/58	0.111
SAPS II, IQR	33 (24.4–42.3)	78/78	34.9 (28–45.8)	58/58	0.190
SOFA, IQR	4 (3–7)	78/78	5 (4–7)	58/58	0.385
Baseline Respiratory parameters					
PaO_2_/FiO_2_, IQR	140 (87–186)	78/78	116 (80–176)	58/58	0.353
Invasive ventilation (%)	59 (77)	77/78	47 (81)	58/58	0.537
Minute ventilation (L/min), IQR	10.4 (9.3–11)	61/78	10.4 (9.7–11.8)	44/58	0.257
PEEP (cm H_2_O), IQR	12 (10–14)	70/78	12 (10–14)	55/58	0.417
PaO_2_ (mmHg), IQR	77 (65–97)	77/78	79 (71–98)	58/58	0.565
PaCO_2_ (mmHg), IQR	40 (34–45)	78/78	37 (33–42)	58/58	0.047
SaO_2_ (%), IQR	96 (94–98)	78/78	97 (94–98)	58/58	0.357
pH_a_, IQR	7.42 (7.38–7.47)	78/78	7.44 (7.4–7.48)	58/58	0.152
Baseline Hemodynamic Parameters					
Norepinephrine use at first PvaCO_2_ assessment (%)	11 (15)	75/78	9 (16)	56/58	0.825
Maximum dose of norepinephrine (µg/kg/min), IQR	0.1 (0.04–0.2)	42/78	0.1 (0.07–0.18)	35/58	0.548
Dobutamine use at first PvaCO_2_ assessment (%)	3 (4)	77/78	3 (5)	58/58	1.000
Central venous pressure (mmHg), IQR	6.5 (2.2–10)	26/78	4 (2.5–9)	11/58	0.738
Baseline Metabolic Parameters					
ScvO_2_ (%), IQR	74.3 (67.6–80)	78/78	69 (64–76.8)	58/58	0.014
Lactate (mmol/L), IQR	1 (0.8–1.4)	77/78	1.1 (0.8–1.4)	58/58	0.512
PvaCO_2_ (mmHg), IQR	5 (4–6)	78/78	8 (7–9)	58/58	<0.001
PvaCO_2_/CavO_2_, IQR	1.49 (1.13–1.8)	76/78	1.98 (1.56–2.28)	57/58	<0.001
Baseline Laboratory values					
Hemoglobin (g/dL), IQR	11.9 (10.5–12.8)	76/78	12.2 (11–13.1)	57/58	0.400
Platelets (10^3^/mm^3^), IQR	228 (193–302)	77/78	223 (171–283)	57/58	0.332
D-dimer (ng/mL), IQR	1000 (613–1903)	37/78	1306 (680–2610)	30/58	0.553
CRP (mg/L), IQR	178 (100–255)	77/78	163 (92–226)	57/58	0.275

Values are median (IQR) or n (%). Available n/N indicates the number of observations available for analysis relative to the total number of patients in each PvaCO_2_ group. Analyses were performed using available observations; no imputation of missing data was performed. Continuous variables were compared using the Wilcoxon rank-sum test; categorical variables were compared using Fisher’s exact test. CRP: C-reactive protein; PaO_2_: arterial oxygen partial pressure; PaCO_2_: arterial carbon dioxide partial pressure; PvaCO_2_: central venous-to-arterial carbon dioxide tension difference; PEEP: positive end-expiratory pressure; SAPS II: Simplified Acute Physiology Score II; SOFA: Sequential Organ Failure Assessment; ScvO_2_: central venous oxygen saturation; CavO_2_: arterial-to-central venous oxygen content difference. Demographic characteristics refer to ICU admission. SAPS II and SOFA scores were calculated using the worst values recorded during the first 24 h after ICU admission. Unless otherwise specified, physiological variables were obtained from the closest available measurement within 1 h of the first PvaCO_2_ assessment; paired blood-gas variables and hemoglobin were measured concomitantly. Platelet count, CRP and D-dimer were obtained on the same calendar day as the first PvaCO_2_ assessment. Norepinephrine and dobutamine use refer to the day of the first PvaCO_2_ assessment. Maximum norepinephrine dose refers to the highest dose recorded during the first 5 ICU days among patients who received norepinephrine; patients with no norepinephrine exposure (dose = 0 µg/kg/min) were not included in this analysis. For the maximum-dose variable, the reported n/N therefore represents the number of patients included in the dose analysis rather than simple data availability.

## Data Availability

The data supporting the findings of this study are not publicly available because of ethical and data-protection restrictions concerning patient-level data. De-identified data may be made available by the corresponding author upon reasonable request, subject to approval by the relevant ethics committee and applicable institutional and data-protection requirements.

## Supplementary Materials

The following supporting information can be downloaded at: https://www.mdpi.com/article/10.3390/diagnostics16213522/s1. Figure S1. Flow chart of the patients enrolled in the study; Figure S2. Cumulative incidence of 90-day ICU outcomes according to baseline PvaCO_2_ group. The figure shows stacked state probabilities over 90 days from ICU admission for patients with low PvaCO_2_ (≤6 mmHg; n = 78; ICU deaths = 23) and high PvaCO_2_ (>6 mmHg; n = 58; ICU deaths = 23). Red areas represent ICU death, green areas alive ICU discharge, and grey areas remaining in the ICU. The dashed horizontal lines indicate the maximum cumulative probability reached for ICU death and ICU discharge in each panel; Figure S3. Distribution of venous-to-arterial carbon dioxide tension (PvaCO_2_) values in patients with mild, moderate and severe hypoxemia according to the ratio of arterial oxygen partial pressure (PaO2 in mmHg) to fractional inspired oxygen (FiO2); Figure S4. Distribution of patients according to central venous oxygen saturation (ScvO2) and venous-to-arterial carbon dioxide tension (PvaCO_2_) at the first evaluation after ICU admission; Figure S5. Longitudinal fixed-effect estimates of physiological parameters in patients with normal baseline veno-arterial carbon dioxide difference (PvaCO_2_ ≤ 6 mmHg), stratified by outcome (alive vs. deceased); Figure S6. Longitudinal fixed-effect estimates of physiological parameters in patients with abnormal baseline veno-arterial carbon dioxide difference (PvaCO_2_ > 6 mmHg), stratified by outcome (alive vs. deceased); Table S1. Primary and sensitivity analyses of the association between PvaCO_2_ and 28-day ICU mortality; Table S2. Bayesian sensitivity analysis of the association between PvaCO_2_ and ICU death within 28 days; Table S3. Interaction analyses between PvaCO_2_ and hypoxemia severity for 28-day ICU mortality; Table S4. Interaction analyses between PvaCO_2_ and ScvO_2_ for 28-day ICU mortality; Table S5 Availability of serial PvaCO_2_ measurements according to baseline PvaCO_2_ category and 28-day ICU outcome.

## References

[1] Teboul J.L., Mercat A., Lenique F., Berton C., Richard C. (1998). Value of the venous-arterial PCO_2_ gradient to reflect the oxygen supply to demand in humans: Effects of dobutamine. Crit. Care Med..

[2] Mecher C.E., Rackow E.C., Astiz M.E., Weil M.H. (1990). Venous hypercarbia associated with severe sepsis and systemic hypoperfusion. Crit. Care Med..

[3] Bakker J., Vincent J.L., Gris P., Leon M., Coffernils M., Kahn R.J. (1992). Veno-arterial carbon dioxide gradient in human septic shock. Chest.

[4] Ltaief Z., Schneider A.G., Liaudet L. (2021). Pathophysiology and clinical implications of the veno-arterial PCO_2_ gap. Crit. Care.

[5] Mallat J. (2016). Use of venous-to-arterial carbon dioxide tension difference to guide resuscitation therapy in septic shock. World J. Crit. Care Med..

[6] Shastri L., Kjærgaard B., Rees S.E., Thomsen L.P. (2021). Changes in central venous to arterial carbon dioxide gap (PCO_2_ gap) in response to acute changes in ventilation. BMJ Open Respir. Res..

[7] Luft U.C., Mostyn E.M., Loeppky J.A., Venters M.D. (1981). Contribution of the Haldane effect to the rise of arterial Pco_2_ in hypoxic patients breathing oxygen. Crit. Care Med..

[8] Italia L., Tomasoni D., Bisegna S., Pancaldi E., Stretti L., Adamo M., Metra M. (2021). COVID-19 and Heart Failure: From Epidemiology During the Pandemic to Myocardial Injury, Myocarditis, and Heart Failure Sequelae. Front. Cardiovasc. Med..

[9] Chiumello D., Busana M., Coppola S., Romitti F., Formenti P., Bonifazi M., Pozzi T., Palumbo M.M., Cressoni M., Herrmann P. (2020). Physiological and quantitative CT-scan characterization of COVID-19 and typical ARDS: A matched cohort study. Intensive Care Med..

[10] Grasselli G., Tonetti T., Protti A., Langer T., Girardis M., Bellani G., Laffey J., Carrafiello G., Carsana L., Rizzuto C. (2020). Pathophysiology of COVID-19-associated acute respiratory distress syndrome: A multicentre prospective observational study. Lancet Respir. Med..

[11] Ospina-Tascón G.A., De Backer D., Aldana J.L., Marín A.F.G., Calderón L.E., Chica J., García-Gallardo G., Orozco N., Mallat J. (2025). Regional venous-to-arterial carbon dioxide pressure and content differences during endotoxemic shock: Influence of hydrogen ion accumulation vs. Haldane effect. Intensive Care Med. Exp..

[12] Troskot R., Šimurina T., Žižak M., Majstorović K., Marinac I., Mrakovčić-Šutić I. (2010). Prognostic value of venoarterial carbon dioxide gradient in patients with severe sepsis and septic shock. Croat. Med. J..

[13] Du W., Liu D.W., Wang X.T., Long Y., Chai W.Z., Zhou X., Rui X. (2013). Combining central venous-to-arterial partial pressure of carbon dioxide difference and central venous oxygen saturation to guide resuscitation in septic shock. J. Crit. Care.

[14] Mallat J., Pepy F., Lemyze M., Gasan G., Vangrunderbeeck N., Tronchon L., Vallet B., Thevenin D. (2014). Central venous-to-arterial carbon dioxide partial pressure difference in early resuscitation from septic shock: A prospective observational study. Eur. J. Anaesthesiol..

[15] Helmy T., El-reweny E., Ghazy F. (2017). Prognostic value of venous to arterial carbon dioxide difference during early resuscitation in critically ill patients with septic shock. Indian J. Crit. Care Med..

[16] Zante B., Reichenspurner H., Kubik M., Schefold J.C., Kluge S. (2019). Increased admission central venous-arterial CO_2_ difference predicts ICU-mortality in adult cardiac surgery patients. Heart Lung.

[17] Shaban M., Salahuddin N., Kolko M.R., Sharshir M., AbuRageila M., AlHussain A. (2017). The Predictive Ability of PV-ACO_2_ Gap and PV-ACO2/CA-VO2 Ratio in Shock. Shock.

[18] Chen T., Pan T., Luo X., Wang D. (2019). High Central Venous-to-Arterial CO_2_ Difference is Associated with Poor Outcomes in Patients after Cardiac Surgery: A Propensity Score Analysis. Shock.

[19] Yang X., Zhou Y., Liu A., Pu Z. (2022). Relationship between Dynamic Changes of Microcirculation Flow, Tissue Perfusion Parameters, and Lactate Level and Mortality of Septic Shock in ICU. Contrast Media Mol. Imaging.

[20] Araujo D.T., Felice V.B., Meregalli A.F. (2019). Value of central venous to arterial CO_2_ difference after early goal-directed therapy in septic shock patients. Indian J. Crit. Care Med..

[21] Sindhu K., Malviya D., Parashar S., Pandey C., Nath S.S., Misra S. (2022). Correlation of central venous-to-arterial carbon dioxide difference to arterial-central venous oxygen difference ratio to lactate clearance and prognosis in patients with septic shock: A prospective observational cohort study. Int. J. Crit. Illn. Inj. Sci..

[22] Lundin A., Annborn M., Borgquist O., Düring J., Undén J., Rylander C. (2023). Veno-arterial CO_2_ difference and lactate for prediction of early mortality after cardiac arrest. Acta Anaesthesiol. Scand..

[23] Scherr B.F., Wendel-Garcia P.D., Bruns H., Steiger P., Schuepbach R.A., Kim B.S., Vasella M., Camen G., Buehler P.K. (2025). Predictive value of veno-arterial carbon dioxide partial pressure difference and central venous oxygen saturation for fluid administration and mortality in critically ill patients with extensive burns. Burns.

[24] Ospina-Tascón G.A., Bautista-Rincón D.F., Umaña M., Tafur J.D., Gutiérrez A., García A.F., García A.F., Bermúdez W., Granados M., Arango-Dávila C. (2013). Persistently high venous-to-arterial carbon dioxide differences during early resuscitation are associated with poor outcomes in septic shock. Crit. Care.

[25] Van Beest P.A., Lont M.C., Holman N.D., Loef B., Kuiper M.A., Boerma E.C. (2013). Central venous-arterial pCO_2_ difference as a tool in resuscitation of septic patients. Intensive Care Med..

[26] Muller G., Mercier E., Vignon P., Henry-Lagarrigue M., Kamel T., Desachy A., Botoc V., Plantefève G., Frat J., Bellec F. (2017). Prognostic significance of central venous-to-arterial carbon dioxide difference during the first 24 hours of septic shock in patients with and without impaired cardiac function. Br. J. Anaesth..

[27] Zirpe K.G., Tiwari A.M., Kulkarni A.P., Vaidya H.S., Gurav S.K., Deshmukh A.M., Suryawanshi P.B., Kapse U.S., Bhoyar A.P., Vaidya H.S. (2024). The Evolution of Central Venous-to-arterial Carbon Dioxide Difference (PCO_2_ Gap) during Resuscitation Affects ICU Outcomes: A Prospective Observational Study. Indian J. Crit. Care Med..

[28] Mallat J., Jozwiak M., Orozco N., Hamzaoui O., Monnet X., Teboul J.L., De Backer D., Ospina-Tascón G.A. (2025). Use of CO_2_-derived variables in critically ill patients. Ann. Intensive Care.

[29] Squara P. (2014). Central venous oxygenation: When physiology explains apparent discrepancies. Crit. Care.

[30] Vallée F., Vallet B., Mathe O., Parraguette J., Mari A., Silva S., Samii K., Fourcade O., Genestal M. (2008). Central venous-to-arterial carbon dioxide difference: An additional target for goal-directed therapy in septic shock?. Intensive Care Med..

[31] Robin E., Futier E., Pires O., Fleyfel M., Tavernier B., Lebuffe G., Vallet B. (2015). Central venous-to-arterial carbon dioxide difference as a prognostic tool in high-risk surgical patients. Crit. Care.

