# Supplementary material for: Prognostic Value of the Veno-Arterial Carbon Dioxide Difference in COVID-19-Associated Acute Hypoxemic Respiratory Failure: A Retrospective Cohort Study
Source: Diagnostics (Basel). 2026 Oct 27;16(21):3522. doi: 10.3390/diagnostics16213522 (PMC13762414; doi:10.3390/diagnostics16213522)
Supplement: Supplementary file 1 [file diagnostics-16-03522-s001.zip › diagnostics-4574212-supplementary.pdf]

Prognostic Value of the Veno-Arterial Carbon Dioxide Difference in COVID-19-Associated Acute Hypoxemic Respiratory Failure: A Retrospective Cohort Study

Keitiane Kaefer, Sara Fratino, Rocio Rimachi, Antonella Cudia, Rachid Attou, Pierre Foulon, Charalampos Pierrakos and Daniel De Backer

Supplementary material

Supplementary Figure S1. Flow chart of the patients enrolled in the study

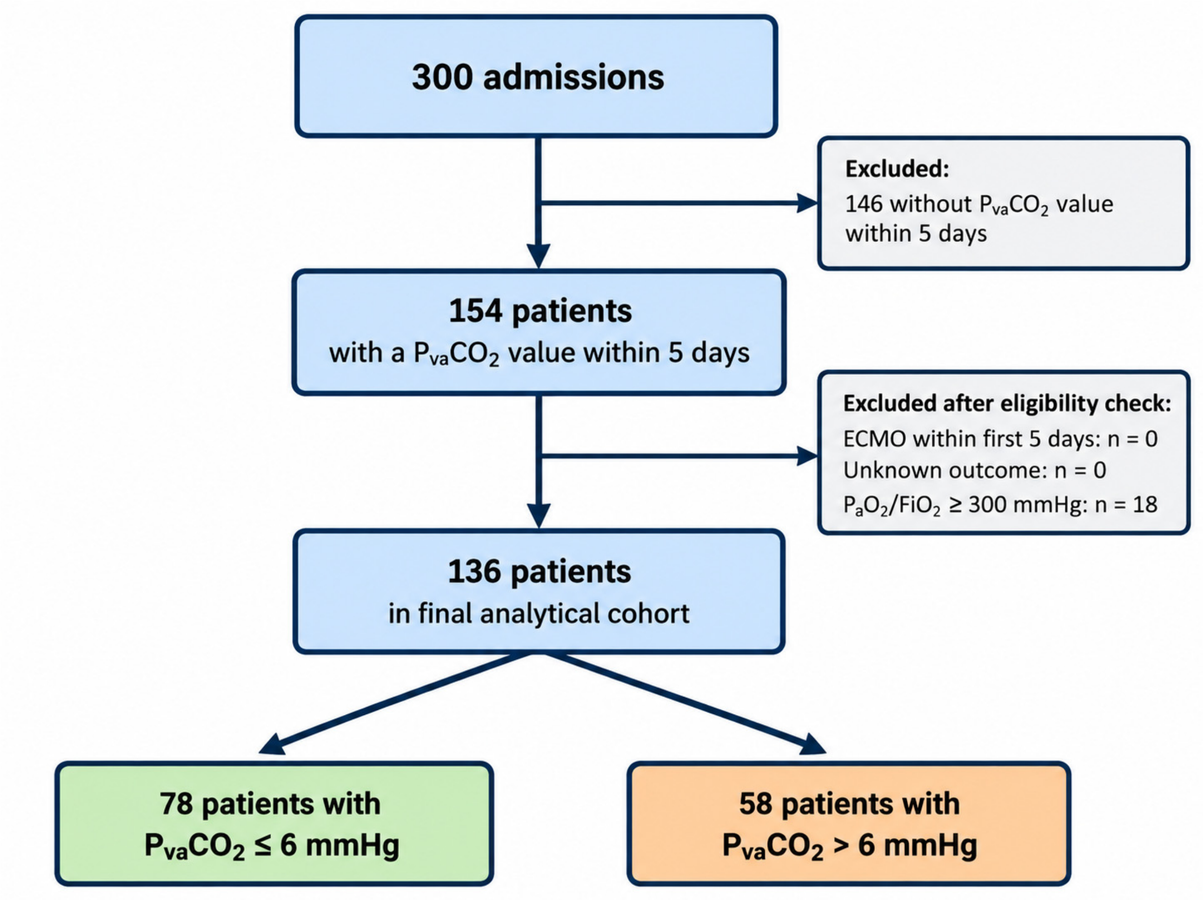

Supplementary Figure S2. Cumulative incidence of 90-day ICU outcomes according to baseline $PvaCO_2$ group. The figure shows stacked state probabilities over 90 days from ICU admission for patients with low $PvaCO_2$ (≤6 mmHg; n = 78; ICU deaths = 23) and high $PvaCO_2$ (>6 mmHg; n = 58; ICU deaths = 23). Red areas represent ICU death, green areas alive ICU discharge, and grey areas remaining in the ICU. The dashed horizontal lines indicate the maximum cumulative probability reached for ICU death and ICU discharge in each panel.

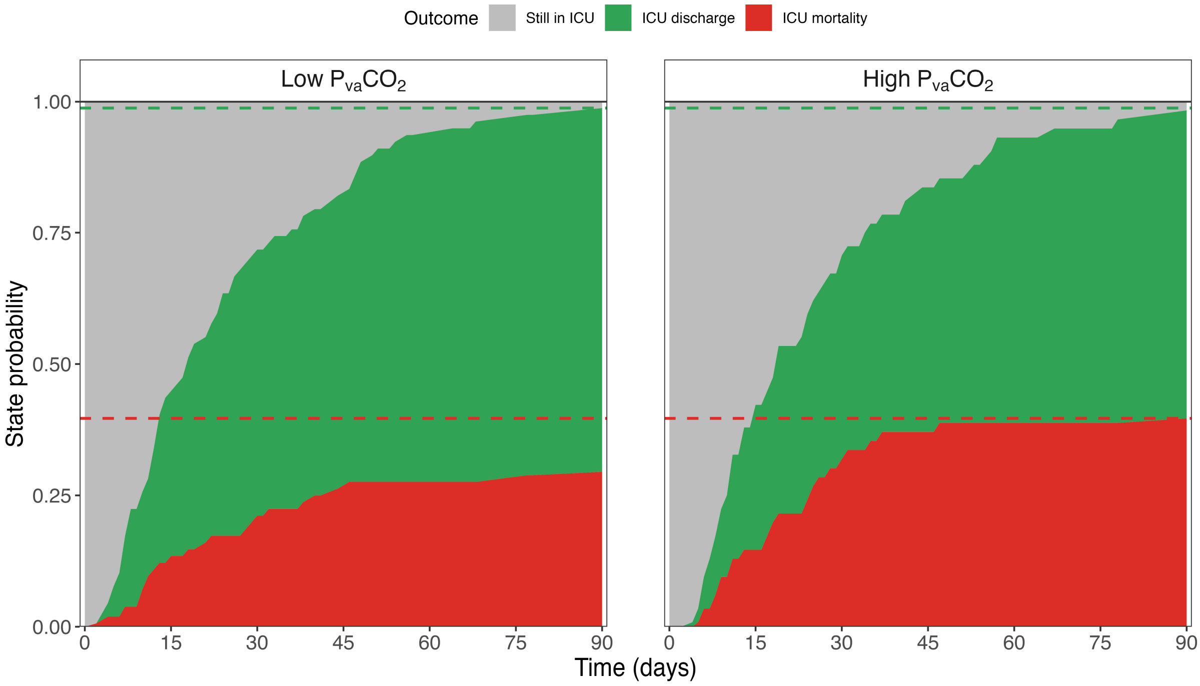

Supplementary Figure S3. Distribution of venous-to-arterial carbon dioxide tension ($P_{va}CO_2$) values in patients with mild, moderate and severe hypoxemia according to the ratio of arterial oxygen partial pressure (P_aO_2 in mmHg) to fractional inspired oxygen (FiO_2)

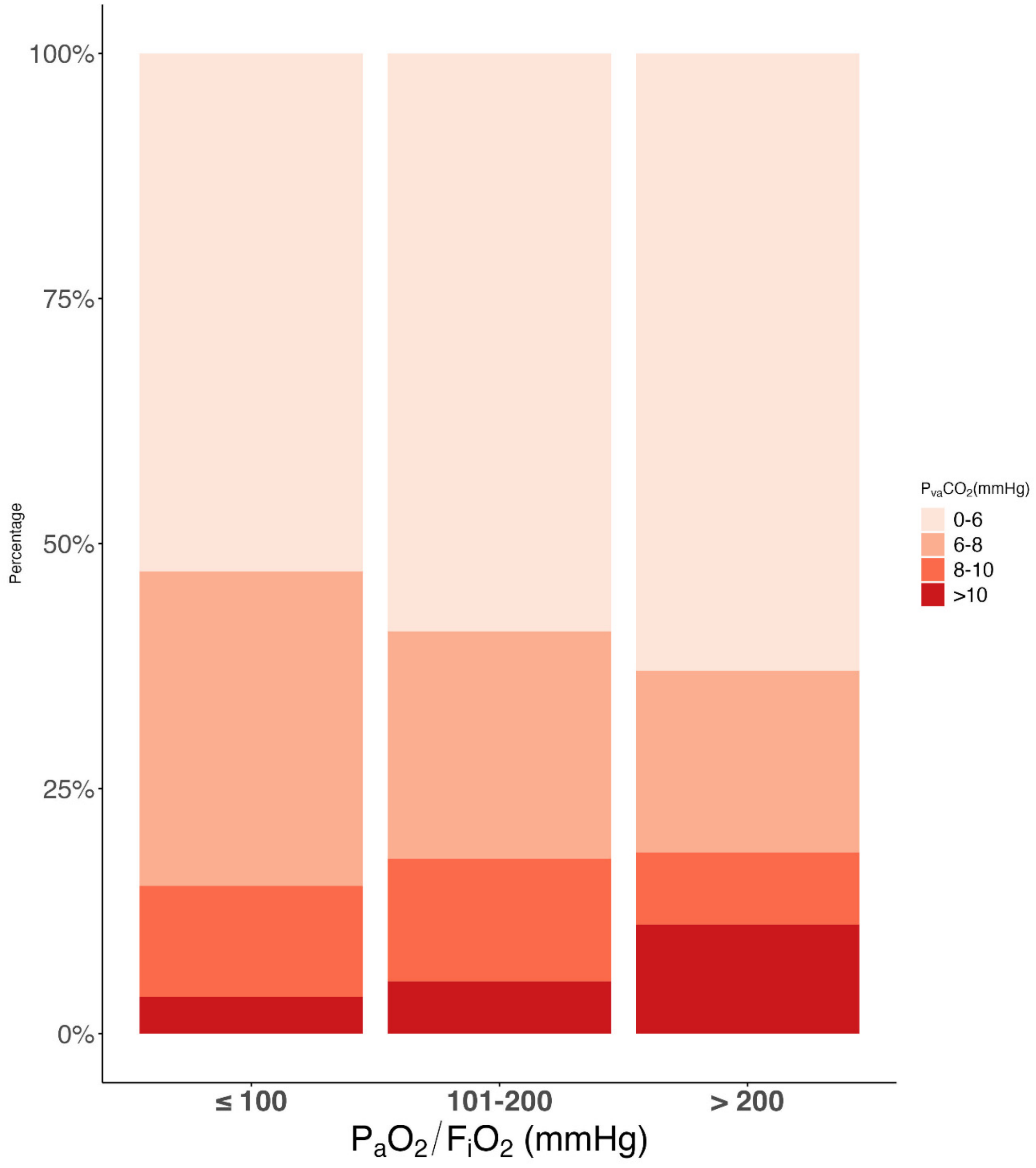

Supplementary Figure S4. Distribution of patients according to central venous oxygen saturation (ScvO2) and venous-to-arterial carbon dioxide tension (PvaCO2) at the first evaluation after ICU admission.

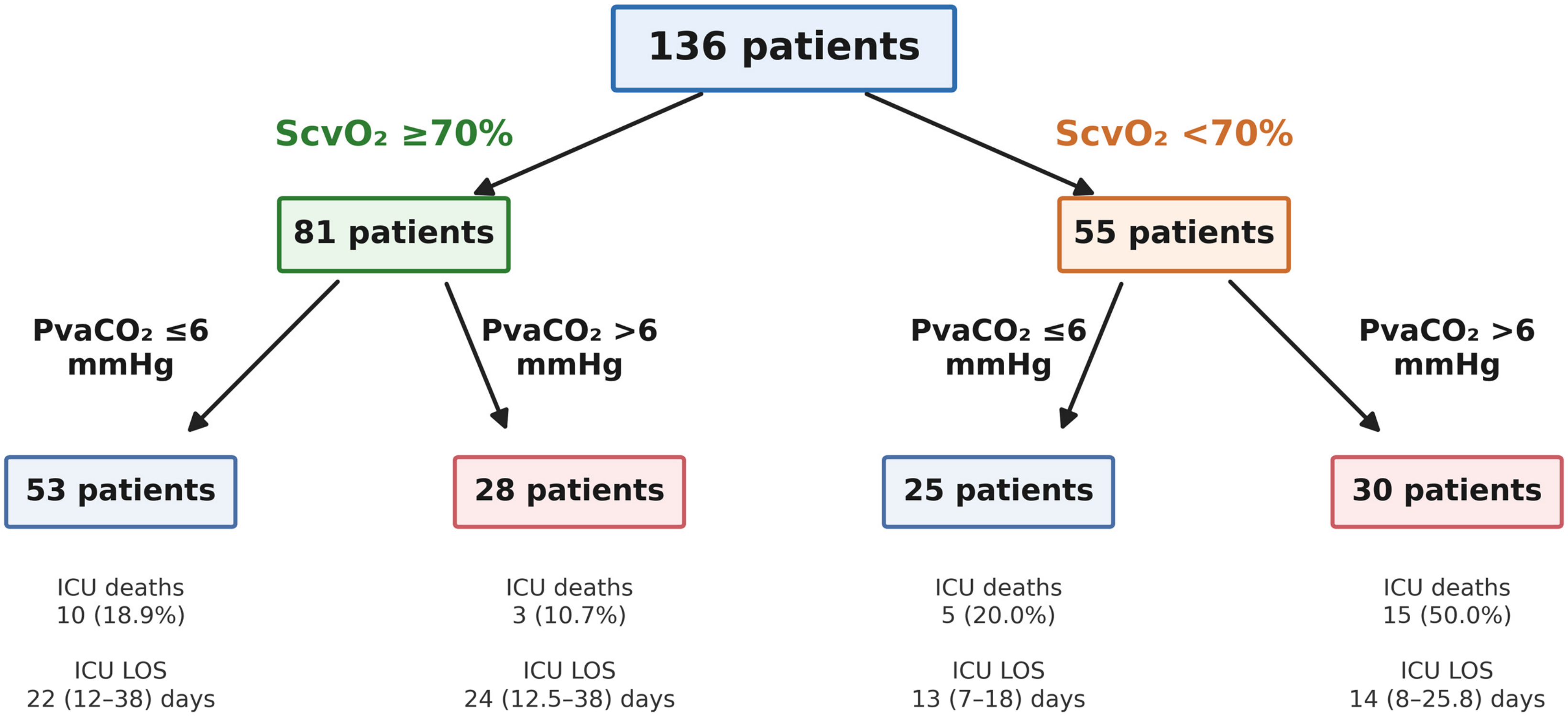

Supplementary Figure S5. Longitudinal fixed-effect estimates of physiological parameters in patients with normal baseline veno-arterial carbon dioxide difference (PvaCO2 ≤ 6 mmHg), stratified by outcome (alive vs. deceased).

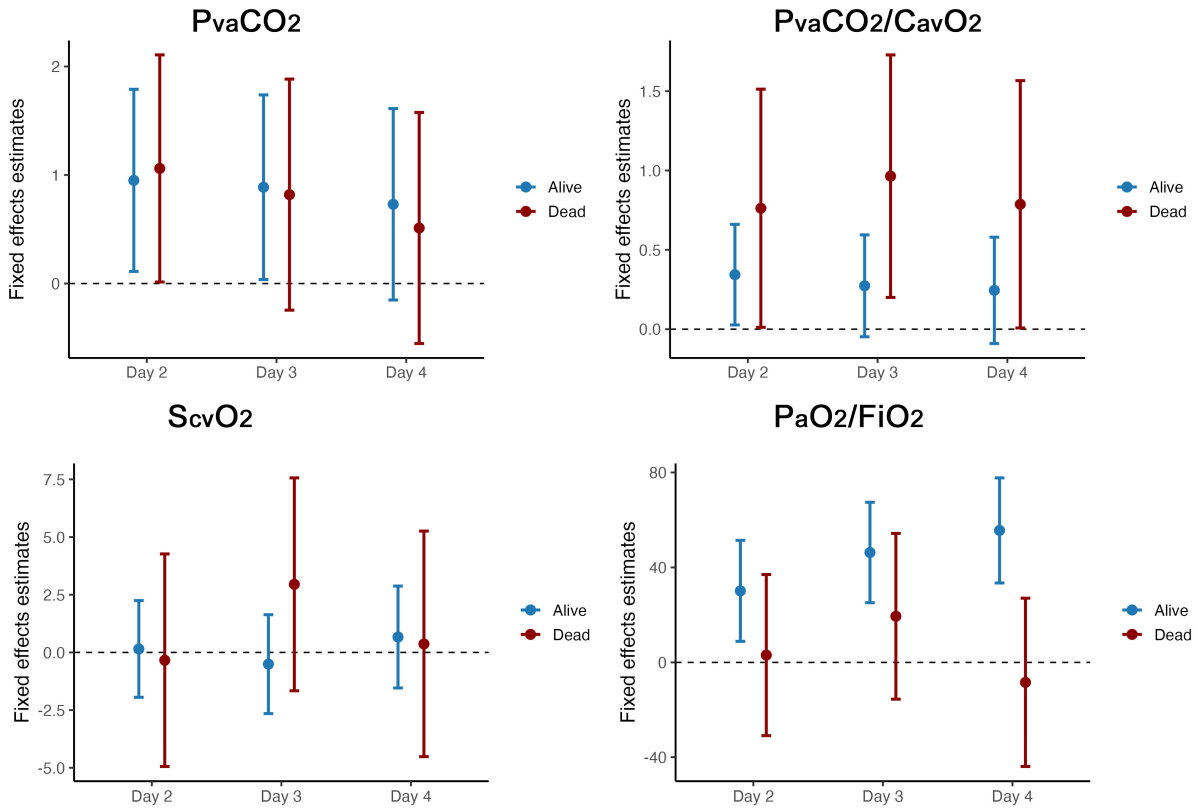

Supplementary Figure S6. Longitudinal fixed-effect estimates of physiological parameters in patients with abnormal baseline veno-arterial carbon dioxide difference (PvaCO2 > 6 mmHg), stratified by outcome (alive vs. deceased).

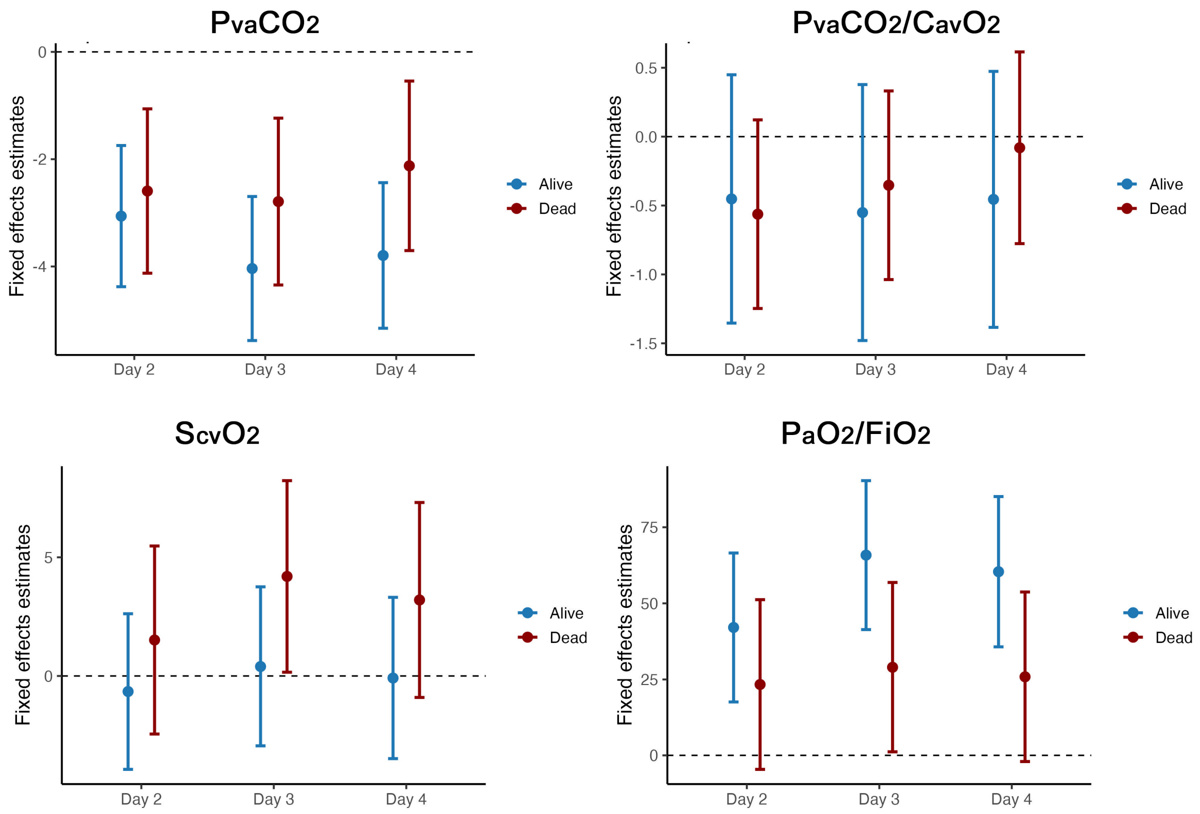

Supplementary Table S1. Primary and sensitivity analyses of the association between $P_{va}CO_2$ and 28-day ICU mortality

Model	Time origin / population	n	ICU deaths	sHR for high vs low $P_{va}CO_2$ (95% CI)	P value
Unadjusted Fine–Gray	ICU admission (main analysis)	136	33	1.69 (0.86–3.33)	0.130
	First $P_{va}CO_2$ assessment	136	34	1.77 (0.91–3.46)	0.095
	First $P_{va}CO_2$ assessment ≤48 h	103	25	2.35 (1.04–5.30)	0.041
Adjusted for SAPS II	ICU admission	136	33	1.39 (0.71–2.75)	0.340
	First $P_{va}CO_2$ assessment	136	34	1.46 (0.74–2.87)	0.270
	First $P_{va}CO_2$ assessment ≤48 h	103	25	1.87 (0.84–4.16)	0.130
Adjusted for study centre	ICU admission	136	33	1.61 (0.82–3.15)	0.170
	First $P_{va}CO_2$ assessment	136	34	1.67 (0.86–3.26)	0.130
	First $P_{va}CO_2$ assessment ≤48 h	103	25	2.17 (0.96–4.90)	0.062
Adjusted for SAPS II + study centre	ICU admission	136	33	1.39 (0.70–2.75)	0.340
	First $P_{va}CO_2$ assessment	136	34	1.46 (0.74–2.85)	0.275
	First $P_{va}CO_2$ assessment ≤48 h	103	25	1.88 (0.85–4.17)	0.120

Notes. All analyses used Fine–Gray competing-risk regression, with ICU death as the event of interest and alive ICU discharge as the competing event. The main analysis used ICU admission as time zero and assessed events through day 28. Analyses using the first $P_{va}CO_2$ assessment as time zero assessed events during the subsequent 28 days. The ≤48-h analyses retained ICU admission as time zero and assessed events through day 28 after ICU admission. Adjusted models included SAPS II alone, study centre alone, or both covariates, as indicated. Only the $P_{va}CO_2$ effect estimate is shown.

Abbreviations: ICU, intensive care unit; sHR, subdistribution hazard ratio; CI, confidence interval; SAPS II, Simplified Acute Physiology Score II.

Supplementary Table S2. Bayesian sensitivity analysis of the association between $PvaCO_2$ and ICU death within 28 days

Prior for log-HR	Posterior median HR (95% CrI)	P(HR > 1)	Rhat	Bulk ESS	Tail ESS
Normal(0, 5)	1.55 (0.78–3.08)	89.7%	1.00	9497	8091
Normal(0, 1)	1.47 (0.77–2.81)	88.0%	1.00	9215	7815
Normal(0, 0.5)	1.35 (0.77–2.35)	85.3%	1.00	9806	8486

Notes. Bayesian Cox proportional-hazards models were fitted in 136 patients, with ICU admission as time zero and ICU death within 28 days as the outcome. High $PvaCO_2$ was compared with low $PvaCO_2$. Priors were specified on the log-hazard-ratio scale. P(HR > 1) denotes the posterior probability that high $PvaCO_2$ is associated with a higher hazard of ICU death within 28 days. All models used four chains with 4,000 iterations per chain, including 1,000 warm-up iterations, yielding 12,000 post-warm-up draws. All models showed satisfactory convergence (Rhat = 1.00), with high bulk and tail effective sample sizes, no divergent transitions, and no maximum-treedepth events. Trace plots showed satisfactory mixing. The proportional-hazards assumption showed no evidence of violation (Schoenfeld residual test, P = 0.47).

Abbreviations. HR, hazard ratio; CrI, credible interval; ESS, effective sample size; ICU, intensive care unit; Rhat, potential scale reduction factor.

Supplementary table S3. Interaction analyses between $PvaCO_2$ and hypoxemia severity for 28-day ICU mortality

Analysis	Hypoxemia stratum	n	ICU deaths	sHR (95% CI)	P value	Pinteraction
Categorical $PvaCO_2$ High vs Low	Mild PaO_2/FiO_2 >200	26	5	0.44 (0.05–3.86)	0.457	0.075
	Moderate PaO_2/FiO_2 101–200	56	10	6.68 (1.36–32.69)	0.019	
	Severe PaO_2/FiO_2 ≤100	54	18	1.06 (0.44–2.57)	0.902	
Continuous $PvaCO_2$ per +1 mmHg	Mild PaO_2/FiO_2 >200	26	5	1.01 (0.83–1.22)	0.928	0.204
	Moderate PaO_2/FiO_2 101–200	56	10	1.24 (0.95–1.63)	0.112	
	Severe PaO_2/FiO_2 ≤100	54	18	0.93 (0.79–1.10)	0.410	
Continuous $PvaCO_2$ × continuous PaO_2/FiO_2	Continuous PaO_2/FiO_2 (per +50 mmHg)	136	33	1.012 (0.953–1.076)	—	0.692

Notes. Subdistribution hazard ratios (sHRs) were estimated using Fine–Gray competing-risk regression, with ICU death by day 28 as the event of interest and alive ICU discharge as the competing event. Hypoxemia strata were defined as mild (PaO_2/FiO_2 >200 mmHg), moderate (PaO_2/FiO_2 101–200 mmHg), and severe (PaO_2/FiO_2 ≤100 mmHg). For categorical $PvaCO_2$, sHRs compare high versus low $PvaCO_2$ within each hypoxemia stratum. For continuous $PvaCO_2$, sHRs are reported per 1-mmHg increase. Global Pinteraction values for categorical hypoxemia were obtained from 2-df Wald tests. In the continuous PaO_2/FiO_2 interaction model, PaO_2/FiO_2 was scaled per 50-mmHg increase; the interaction estimate is the multiplicative change in the $PvaCO_2$ sHR per 50-mmHg increase in PaO_2/FiO_2.

Abbreviations: ICU, intensive care unit; sHR, subdistribution hazard ratio; CI, confidence interval; PaO_2/FiO_2, arterial oxygen partial pressure to inspired oxygen fraction ratio.

Supplementary table S4. Interaction analyses between $PvaCO_2$ and $ScvO_2$ for 28-day ICU mortality

Analysis	$ScvO_2$ stratum	n	ICU deaths	sHR (95% CI)	P value	Pinteraction
Categorical $PvaCO_2$ High vs Low	High $ScvO_2$ ≥70%	81	13	0.54 (0.15–1.93)	0.341	0.044
	Low $ScvO_2$ <70%	55	20	2.89 (1.04–7.98)	0.041	
Continuous $PvaCO_2$ per +1 mmHg	High $ScvO_2$ ≥70%	81	13	0.98 (0.82–1.18)	0.860	0.471
	Low $ScvO_2$ <70%	55	20	1.11 (0.85–1.43)	0.448	
Continuous $PvaCO_2$ × continuous $ScvO_2$	Continuous $ScvO_2$ (per +5 percentage points)	136	33	1.008 (0.956–1.062)	—	0.778

Notes. Subdistribution hazard ratios (sHRs) were estimated using Fine–Gray competing-risk regression, with ICU death by day 28 as the event of interest and alive ICU discharge as the competing event. $ScvO_2$ was categorized as high (≥70%) or low (<70%). For categorical $PvaCO_2$, sHRs compare high versus low $PvaCO_2$ within each $ScvO_2$ stratum. For continuous $PvaCO_2$, sHRs are reported per 1-mmHg increase. Pinteraction for categorical $ScvO_2$ corresponds to the 1-df Wald test of the $PvaCO_2$ × $ScvO_2$ interaction. In the continuous $ScvO_2$ interaction model, $ScvO_2$ was scaled per 5-percentage-point increase; the interaction estimate represents the multiplicative change in the $PvaCO_2$ sHR per 5-percentage-point increase in $ScvO_2$.

Abbreviations: ICU, intensive care unit; sHR, subdistribution hazard ratio; CI, confidence interval; $ScvO_2$, central venous oxygen saturation.

Supplementary Table S5. Availability of serial $P_{va}CO_2$ measurements according to baseline $P_{va}CO_2$ category and 28-day ICU outcome

Baseline $P_{va}CO_2$	28-day ICU outcome	Study day	Available measurements, n (%)	ICU discharge, n	ICU death, n	Missing while still in ICU, n
≤6 mmHg	Alive (N=63)	Day 1	63 (100.0)	0	0	0
		Day 2	55 (87.3)	1	0	7
		Day 3	52 (82.5)	2	0	9
		Day 4	47 (74.6)	4	0	12
	Deceased (N=15)	Day 1	15 (100.0)	0	0	0
		Day 2	10 (66.7)	0	1	4
		Day 3	10 (66.7)	0	1	4
		Day 4	10 (66.7)	0	1	4
>6 mmHg	Alive (N=40)	Day 1	40 (100.0)	0	0	0
		Day 2	33 (82.5)	0	0	7
		Day 3	31 (77.5)	0	0	9
		Day 4	30 (75.0)	0	0	10
	Deceased (N=18)	Day 1	18 (100.0)	0	0	0
		Day 2	16 (88.9)	0	0	2
		Day 3	15 (83.3)	0	0	3
		Day 4	14 (77.8)	0	0	4

Note. Percentages for available measurements are calculated using the baseline number of patients in each stratum as the denominator. “ICU discharge” and “ICU death” indicate that the patient was no longer available for longitudinal assessment by the corresponding study day. “Missing while still in ICU” indicates absence of a $P_{va}CO_2$ measurement despite the patient remaining under ICU observation.

Abbreviation: $P_{va}CO_2$, venous-to-arterial carbon dioxide partial pressure difference.
